# Factors for suicide attempt recurrence among patients with borderline personality disorder

**DOI:** 10.1192/j.eurpsy.2021.1561

**Published:** 2021-08-13

**Authors:** S. Brahim, M.H. Aoun, O. Charaa, M. Henia, A. Abid, L. Zarrouk

**Affiliations:** 1 Psychiatry, University Hospital of Mahdia, Tunisia., chebba, Tunisia; 2 Psychiatry, University Hospital Of Mahdia., Mahdia, Tunisia; 3 Department Of Psychiatry, University Hospital Of Mahdia, Tunisia., Psychiatry, Mahdia, Tunisia; 4 Anesthesia, University Hospital of Mahdia, Tunisia., mahdia, Tunisia

**Keywords:** Borderline personality disorder, Suicide, prevalence, predictive factors

## Abstract

**Introduction:**

The prevalence of borderline personality disorder (BPD) is significant, ranging from 0.5% to 5.9% in the general population. This personality disorder is associated with high rates of suicide attempt and for suicide attempt recurrence.

**Objectives:**

Review recent studies of predictors of suicide attempt and for suicide attempt recurrence in patients with borderline personality disorder.

**Methods:**

This is a literature review via Medline and Sciences Direct. The database was searched using the combination of the keywords “borderline” with “suicide”, “borderline personality disorder” with “suicide”, “borderline personality disorder” with “suicide attempts” “borderline personality disorder” with “suicide recurrence”.

**Results:**

Recently it has been shown that BPD has a greater correlation with suicidal behavior than that of characterized depressive disorders. Several studies have shown that suicidal behavior in patients with BPD was associated with the coexistence of antisocial personality disorder, depression, hostility, impulsivity, a high number of suicide attempts and a first suicide attempt at a young age. Recently it has been established that the predictors of suicidal recidivism are the high number of suicide attempts, the female sex and single life status.

**Conclusions:**

Special attention should be paid to predictive factors for suicide attempt and for suicide attempt recurrence in the clinical evaluation of patients with borderline personality disorder, especially in suicidal crisis.

